# A safe haven for cancer cells: tumor plus stroma control by DYRK1B

**DOI:** 10.1038/s41388-025-03275-6

**Published:** 2025-01-25

**Authors:** Miriam Ems, Anna Brichkina, Matthias Lauth

**Affiliations:** 1 Department of Gastroenterology, Endocrinology and Metabolism, Center for Tumor and Immune Biology, Philipps University Marburg, Marburg, Germany; 2 Institute of Systems Immunology, Philipps University Marburg, Marburg, Germany

**Keywords:** Targeted therapies, Oncogenes

## Abstract

The development of resistance remains one of the biggest challenges in clinical cancer patient care and it comprises all treatment modalities from chemotherapy to targeted or immune therapy. In solid malignancies, drug resistance is the result of adaptive processes occurring in cancer cells or the surrounding tumor microenvironment (TME). Future therapy attempts will therefore benefit from targeting both, tumor and stroma compartments and drug targets which affect both sides will be highly appreciated. In this review, we describe a seemingly paradoxical oncogenic mediator with this potential: The dual-specificity tyrosine-phosphorylation regulated kinase 1B (DYRK1B). DYRK1B promotes proliferative quiescence and yet is overexpressed or amplified in many hyperproliferative malignancies including ovarian cancer and pancreatic cancer. In particular the latter disease is a paradigmatic example for a therapy-recalcitrant and highly stroma-rich cancer entity. Here, recent evidence suggests that DYRK1B exerts its oncogenic features by installing a protective niche for cancer cells by directly affecting cancer cells but also the TME. Specifically, DYRK1B not only fosters cell-intrinsic processes like cell survival, chemoresistance, and disease recurrence, but it also upregulates TME and cancer cell-protective innate immune checkpoints and down-modulates anti-tumoral macrophage functionality. In this article, we outline the well-established cell-autonomous roles of DYRK1B and extend its importance to the TME and the control of the tumor immune stroma. In summary, DYRK1B appears as a single novel key player creating a safe haven for cancer cells by acting cell-intrinsically and—extrinsically, leading to the promotion of cancer cell survival, chemoresistance, and relapse. Thus, DYRK1B appears as an attractive drug target for future therapeutic approaches.

## Current cancer therapy concepts

Classical cancer therapy mainly focuses on cytotoxic regiments eradicating the malignant cells of a tumor, however, with limited success. For decades, targeting the epithelial component of carcinomas has left aside the important role of the surrounding TME, presumably explaining why chemotherapeutic drugs often result in initial responses, but are often followed by drug resistance and disease recurrence [1]. On the other hand, solely targeting the TME will also not suffice to combat cancer and some form of additional tumor cell debulking will undoubtedly be necessary. As a result, future treatment approaches for solid cancer types will have to entail both arms, a tumor cell-centric and a TME-based one, and the recent positive experience with immune checkpoint inhibition in combination with chemotherapy e.g., lung cancer supports this view [2]. A drawback of this concept is however the need to target two tumor compartments with two separate drugs, potentiating possible toxicities and reducing their applicability in the clinic. Thus, the identification of single drug targets with the ability to impact both, tumor and stroma, should be of high interest as they would allow modulating both compartments with one drug.

Here, we outline the functions of the DYRK1B kinase and propose its usefulness as a central regulator of the tumor as well as stroma compartments. Small-molecule DYRK inhibitors are now in clinical phase testing and might therefore be soon available for assessment in cancer patients. In our review, we will use pancreatic cancer as an exquisite example for a therapy-refractive TME-rich solid cancer and delineate the cell-intrinsic as well as non-cell-intrinsic roles which predestine the DYRK1B kinase as a promising future drug target.

## Pancreatic cancer: a stroma-rich malignancy

Over 90% of pancreatic cancers are pancreatic ductal adenocarcinomas (PDAC) originating in the exocrine part of the pancreas [3, 4]. Even though the 5-year survival rate of patients suffering from PDAC increased in the US from 1975 (3%) until now, the prognosis for this disease is still the worst compared to other cancer entities (13% in 2024) [5]. Besides being the 12th most common cancer type in humans, pancreatic cancer is the 6th leading cause of cancer-associated death worldwide [6]. With a rising incidence [5, 7], pancreatic cancer is predicted to become the second leading cause of cancer death by 2030 in the US [8]. One reason for the poor prognosis of PDAC is its late diagnosis. Only around 14% of all patients present with a localized tumor, whereas nearly 50% of patients are diagnosed with distant metastatic cancer [9].

Among the modifiable risk factors for PDAC are cigarette smoking and alcohol, but also obesity, diabetes, and low physical activity [10]. Genetic risk factors are among others hereditary pancreatitis and congenital mutations in the DNA damage repair pathway or tumor suppressors like *TP53* [11].

Central to PDAC are activating mutations in the driver gene *KRAS* (Kirsten rat sarcoma virus) with around 90% of all patients carrying such a mutation [12, 13]. Two broad transcriptional subtypes (basal/classical) could thus far be identified in PDAC with the basal subtype harboring more mesenchymal features, a higher aggressiveness [14–16], as well as an increased *KRAS* gene dosage [17]. Another approach classified the tumor stroma into “normal” and “activated” subtypes, with the latter being associated with poorer outcomes [16].

Activated stroma was found to express genes that are involved in tumor promotion and were associated with tumor-associated macrophages (TAMs) as well as activated fibroblasts, two cell populations frequently encountered in the TME [16, 18, 19]. Cancer-associated fibroblasts (CAFs) can deposit huge amounts of extracellular matrix, resulting in histological desmoplasia characterized by a pathologically stiff tissue environment, hypovascularization, and drug impermeation [20–22]. These processes, together with a plethora of immune-suppressive cells (e.g., TAMs, Myeloid-derived suppressor cells, regulatory T-cells (Tregs)) render the TME an immune-secluded place (“cold” TME) where cancer cells can thrive and which is highly plastic to adapt to external pressure evoked by clinical therapies [23–26]. Unfortunately, attempts to overcome this immune blockade have remained unfruitful in the case of PDAC as have attempts to unspecifically deplete CAFs [27–29]. Taken together, it is of great interest to identify novel drug targets which would impact on the cancer cells as well as the stroma in order to debulk the tumor, block TME-mediated adaptation processes, and to overcome immune blockades. As expected, fewer molecular targets would be preferred to fulfill these tasks in order to reduce drug-associated side effects and toxicities. The DYRK1B kinase might be one of such new candidates.

## Dual-specificity tyrosine-phosphorylation regulated kinases (DYRKs)

Regulation of signal transduction is often controlled by phosphorylation of mediator proteins. Kinases, the enzymatic proteins responsible for phosphorylation, play a key role in signaling processes like differentiation, cell shape, movement, growth, and cell cycle control [30]. Kinases are divided into tyrosine (Y) kinases, serine/threonine (S/T) kinases, or dual-specificity kinases, which are able to phosphorylate both Y- and S/T-residues [31, 32].

In 1995, kinases were divided into five groups [33], which was extended to nine groups in 2002 [34]. One of these groups is the CMGC group, named after cyclin-dependent kinases (CDKs), mitogen-activated protein kinases, glycogen synthase kinases, and CDC-like kinases (CLKs) [33]. CMGC kinases are important regulators of signaling pathways associated with proliferation, differentiation, gene expression, cell cycle regulation, and apoptosis [35]. One subclass within the CMGC group is the DYRK family of kinases.

DYRKs are phylogenetically clustered into two classes: Class I comprises DYRK1A and DYRK1B (a.k.a. MIRK), whereas class II comprises DYRK2, DYRK3 and DYRK4 [34]. As part of the CMGC group, the DYRK family members share a highly conserved catalytic domain with the other members [36]. Characteristic for DYRK kinases is the presence of a DYRK homology (DH) box motif (DDDNxDY), which lies upstream of the catalytic domain and is thought to be required for full kinase activation [37, 38]. The members of class I furthermore contain a proline-, glutamic acid-, serine- and threonine-rich (PEST) motif in the noncatalytic C-terminal region, believed to be responsible for the initiation of fast degradation of the protein [39, 40], whereas class II members harbor an N-terminal autophosphorylation accessory region (NAPA) domain, necessary for catalytic activation [41]. As the name indicates, DYRKs are dual-specificity kinases. They are able to phosphorylate both S-/T- and Y-residues, but the Y-phosphorylation is only used for autophosphorylation [42]. The autophosphorylation of a Y-residue within the activation loop is crucial for a confirmational change from an inactive to an active state and occurs already during protein synthesis [39, 43]. The resulting active protein is thus considered a kinase with S-/T-target specificity.

Since DYRKs phosphorylate proteins involved in proliferation, gene expression, apoptosis, and cell cycle regulation [30, 44], all of which crucial steps in tumor progression [45], it is assumed that DYRKs play an important role in cancer development. In this review, we will focus particularly on the role of DYRK1B as it comprises the family member most studied for its role in cancer. It will be interesting to learn to which extent findings from DYRK1B can be extrapolated to other DYRK family members.

## DYRK1B

### DYRK1B and the cell cycle

**Fig. 1:**
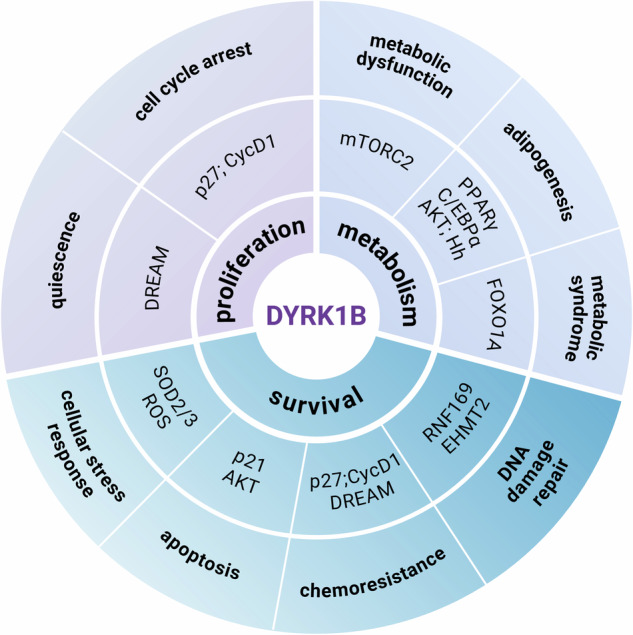
Cell-intrinsic functions of DYRK1B to control proliferation, metabolism, and survival of healthy and neoplastic cells. (Figure created in BioRender.com).

In a healthy organism, the cell cycle is highly regulated and monitored by checkpoints to ensure correct division, and thus proliferation, of cells [46]. Cells can exit the mitotic cycle in G_1_ and enter the G_0_ phase, a state also referred to as quiescence [47]. This non-proliferative state is reversible (in contrast to other non-proliferative states such as senescence) and cells can return to mitosis given the right conditions. In general, cell cycle control can occur in two ways: (i) post-translationally by activation/inactivation of cell cycle regulating proteins and (ii) on the transcriptional level. With respect to the former, DYRK1 kinases negatively influence the cell cycle by phosphorylating Cyclin D and p27^Kip1^, leading to the destabilization and the stabilization of the respective proteins, respectively [48–50]. As Cyclin D binds the CDK 4 and 6, DYRK1 kinases interfere with the phosphorylation of RB proteins [48] and the subsequent release of E2F transcription factors [51, 52]. p27^Kip1^ is a CDK inhibitor, which blocks the phosphorylation of CDK substrates [53]. The DYRK1B-mediated phosphorylation of p27^Kip1^ on Ser10 leads to its stabilization, ultimately resulting in cell cycle arrest at G_0_/G_1_ [48, 50, 54] (Fig. 1).

As mentioned before, proliferative regulation can also occur at the transcriptional level, mediated through the so-called DREAM complex, a multi-protein complex comprised of the dimerization partners of E2F transcription factors, retinoblastoma (RB) proteins, the E2F transcription factors, and the MuvB core. The MuvB core is a stable complex of the proteins RBBP4, LIN9, LIN37, LIN52, and LIN54, which binds to p130, a member of the RB family, during the G_0_/G_1_ phase [55–57]. The DREAM complex associates with gene promotors involved in mitosis progression and thus represses cell cycle-regulated genes during *G*_*0*_ phase [55, 56, 58]. DYRK1 kinases promote DREAM complex assembly by phosphorylating serine 28 of LIN52 [59, 60], thus negatively regulating the cell cycle on a transcriptional level (Fig. 1).

The crucial role of DYRK1B in proliferative control is also reflected in its protein abundance: For instance, DYRK1B levels are increased in non-dividing and differentiating myoblasts [61]. Hence, depleting endogenous DYRK1B with small interfering RNAs can inhibit the differentiation and furthermore allow the cells to re-enter the cell cycle by transitioning from G_0_ into G_1_ phase [50, 61]. According to current knowledge, DYRK1A and DYRK1B appear to act similarly in cell cycle control and pharmacological DYRK1 blockade might thus be exploited to expand pancreatic β-cell pools in the fight against diabetes [62–64] (Fig. 1). The suppressive impact of DYRK1B on the cell cycle cannot only be observed in normal cells, but is also preserved in many cancer cell lines, such as those of ovarian cancer, non-small cell lung cancer, glioblastoma, and PDAC [65–70].

### DYK1B in metabolic control

In normal physiology, DYRK1B plays a crucial role in the differentiation of adipocytes and myoblasts, two important cell populations in metabolic homeostasis [61, 71].

DYRK1B exerts metabolic control by modulating central regulators of adipogenesis and metabolism such as Hedgehog (Hh) signaling and AKT/mTOR pathway activity [72–75] (Fig. 1). Moreover, a recent study showed DYRK1B to be upregulated in patients with nonalcoholic fatty liver disease and insulin resistance [76]. Further investigations led to the conclusion that DYRK1B directly stimulates the activation of mTORC2 (but not mTORC1) in a kinase-independent manner, resulting in an elevated expression of de novo lipogenesis pathway enzymes. Eventually, the increased DYRK1B levels led to hepatic insulin resistance, which could be restored by depletion of DYRK1B [76]. The metabolic impact of loss-of-function *DYRK1B* mutations has also been verified in a human patient cohort [77]. In summary, recent evidence has established DYRK1B as an important novel metabolic regulator and it can be speculated that these characteristics might also be crucial in the setting of malignant disease (Fig. 1).

### DYRK1B and cell-intrinsic survival mechanisms

Even though DYRK1B is a promotor of quiescence, expression of *DYRK1B* is paradoxically elevated in around 40% of pancreatic tumors [78]. Roughly 10% of pancreatic cancer harbor an amplification of the *DYRK1B* gene [79]. Since chemotherapies are often targeting highly proliferating cells, it is assumed that the promotion of quiescence is a mechanism of solid tumors to escape the attack of such treatments [79]. Chemotherapeutic resistance eventually leads to tumor relapse, one of the biggest challenges regarding a successful cancer treatment.

Besides the promotion of cell cycle arrest, DYRK1B was found to protect quiescent cells as well as differentiated myotubes against oxidative stress by increasing the transcription of antioxidant genes like *superoxide dismutase 2* (*SOD2)* and *SOD3*, among others [80, 81]. Additionally, DYRK1B depletion or inhibition in ovarian, lung, and pancreatic cancer cells rendered cells sensitive to chemotherapeutics that induce reactive oxygen species (ROS), such as cisplatin [70, 81–83]. ROS are defined as molecules, which could either be free radicals such as superoxide and hydroxyl radicals, containing an unpaired electron, or non-radical molecules like hydrogen peroxide [84]. ROS arise during normal biological processes such as oxidative phosphorylation in mitochondria, but increased ROS levels may lead to DNA damage and cell death [84, 85] and high DYRK1B levels might thus protect cancer cells (Fig. 1).

Moreover, DYRK1B has been associated with the repair of DNA damage, of which DNA double-strand breaks (DSBs) pose a particular threat to cancer cells. DSBs can occur upon radiation therapy or due to chemotherapeutic agents such as topoisomerase inhibitors [86]. Cells arrest their gene expression temporarily after DSBs, to avoid a collision of the DNA repair machinery and the transcription apparatus [87]. A recent study showed DYRK1B to be involved in the repression of transcription after DNA damage via phosphorylating the histone methyltransferase EHMT2 [88]. Furthermore, they could show that DYRK1B is also required for DSB repair at ribosomal DNA loci by direct interaction with RNF169 to facilitating DYRK1B recruitment at damaged sites [89].

In addition to these protective features, DYRK1B exerts pro-survival functions in myoblasts, rhabdomyosarcoma, colon cancer, as well as in PDAC cells [44, 73, 78, 82, 90–92]. Two molecular mechanisms have been reported to account for this anti-apoptotic outcome: First, DYRK1B can phosphorylate p21 on Ser153, leading to its cytoplasmic accumulation and its presumed inhibitory interaction with pro-apoptotic molecules [90]. Second, DYRK1B overexpression directly or indirectly promotes the activation of the pro-survival AKT protein through induction of Thr308 and Ser473 phosphorylation [73] (Fig. 1).

Eventually, the overall ability of DYRK1B to slow proliferation as well as to foster survival might be one reason why this kinase has previously been linked to cancer stem cells and stem cell-enriched tumor spheroids [93–95]. These findings, together with its additional involvement in cell migration [96, 97] make DYRK1B an attractive future drug target for many cancer entities, especially for those with a genomic *DYRK1B* amplicon as encountered in PDAC and ovarian cancer. In fact, in pancreatic cancer cells, mutant KRAS activates DYRK1B via RAC1, a member of the RHO family [98] and the combinatory depletion of DYRK1B and KRAS led to strongly reduced cancer cell viability [98], which is of high interest in light of recently developed small molecule KRAS inhibitors [99].

### Oncogenic signal transduction and DYRK1B

As described before, DYRK1B plays a crucial role in the activation of mTORC2 [76]. This is in full agreement to DYRK1B’s reported role in AKT activation, a central component of the PI3K/AKT/mTOR pathway. DYRK1B seems to be particularly tightly interwoven with this pathway as several points of interaction exist: (i) DYRK1B overexpression promotes mTOR activation and combined inhibition of DYRK1B and mTOR has superior impact on pancreatic and ovarian cancer cell growth compared to single treatment [67, 73, 94, 100]. As such, mTOR inhibitors could fully abrogate the surplus in cell proliferation upon DYRK1B loss [67]. (ii) The interaction between DYRK1B and mTOR goes in both directions and mTOR signaling can also influence DYRK1B activity. Pharmacological inhibition of mTOR results in upregulation of DYRK1B abundance in PDAC and ovarian cancer cells [94, 100, 101].

The *DYRK1B* gene promotor harbors binding sites for the CREB (cyclic adenosine monophosphate (cAMP) response element binding protein) transcription factor [101]. Inhibition of mTOR increases the activity of CREB and thus potentially the promotor activity of *DYRK1B*, resulting in elevated DYRK1B levels. Furthermore, the inhibition of AMP-activated protein kinase (AMPK), which is an mTOR inhibitor itself, leads to decreased level of phospho-CREB and DYRK1B [101]. However, inhibition of MEK/ERK (which can also phosphorylate CREB) has also been reported to upregulate DYRK1B, raising the question of DYRK1B regulation by hitherto unknown mechanisms of unspecific stress responses [68, 102] (Fig. 1). In conclusion, there seems to be a very close mechanistic interaction between DYRK1B and the mTOR/AKT signaling pathway, which might be very important when considering future therapeutic options. Taken together, literature has accrued numerous evidences for cell-intrinsic roles of DYRK1B in (pancreatic) cancer, affecting signal transduction, proliferation, metabolism, and cell survival.

### DYRK1B and cell-extrinsic mechanisms: the tumor microenvironment

While thus far most of the functions attributed to DYRK1B are intrinsic to tumor cells, the interaction between cancer cells, stromal as well as immune cells significantly influences tumor progression and response to therapy. Solid tumors such as PDAC, lung cancer, and liver cancer often exhibit a highly inflamed stroma and are associated with resistance to chemo- and immunotherapy and short survival [103]. These cancer types are characterized by a potent immunosuppressive TME, typically excluding cytotoxic T cells and NK cells from the tumor site or driving them into functional exhaustion. TAMs are among the most abundant immune cells infiltrating the TME. At later stages of tumor progression, TAMs create an inflammatory immunosuppressive niche that supports cancer growth and contributes to metastasis. Nevertheless, during the earliest stages of tumor onset, the immune system promotes the activation of macrophages to eliminate tumor cells through phagocytosis or cytotoxic killing [104]. The phagocytic capacity of macrophages and their ability to efficiently infiltrate tumors and eliminate tumor cells are being actively exploited as effective cancer immunotherapy strategies [105], including innovative approaches such as designing CAR-modified macrophages [106].

When considering a future clinical use of DYRK inhibitors, systemic therapy would affect both, tumor cells and the TME. Intriguingly, DYRK1B might play a direct role in TAMs. Two studies by Blom et al. [107, 108] demonstrated using the THP-1 cell line that the specific DYRK1B inhibitor AZ191 and the less-specific compound mebendazole shifted the polarization of TAMs from a pro-tumorigenic M2 subtype, expressing CD163, to a more M1-like subtype with enhanced secretion of inflammatory factors such as IL-1β, TNF, IL-6, and CXCL10 [107]. Moreover, mebendazole reduced the growth of HT29 colon cancer cells due to the suppressive effects of THP-1 macrophages. These findings provide the first evidence that inhibition of DYRK1B might target cells in the TME, particularly by reducing the tumor-promoting impact of TAMs. Other findings have been reported for astrocytes and microglial cells where knockdown of DYRK1B reduced LPS-induced neuroinflammation [109] and the DYRK1A/B inhibitor Harmine significantly attenuated microglia activation and neuronal damage via the TLR4/NF-κB signaling pathway [110]. In a model of skin inflammation, inhibition of DYRK1B attenuated inflammatory responses in a murine contact hypersensitivity model of allergic contact dermatitis by suppressing the differentiation of Th1 and Th17 cells in the regional lymph node and enhancing the differentiation of regulatory T cells (Tregs) in vitro [111]. Furthermore, downregulation of DYRK1B by miR-9 enhanced NFAT activity and promoted IL-2 production in Jurkat T cells [112], which potentially can positively impact cytotoxic T cell responses in vivo. Although these data do not provide a direct link to tumor immunity, they collectively demonstrate that pharmaceutical compounds targeting DYRK1B could potentially intervene not only in the cancer cell compartment but also in its microenvironment.

**Fig. 2:**
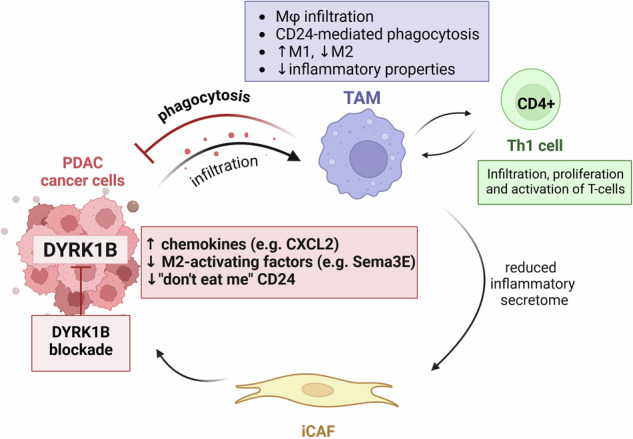
The role of DYRK1B in the tumor microenvironment: cell-extrinsic mechanisms. Tumor-expressed DYRK1B helps shield cancer cells by excluding macrophages from the tumor site and protecting cancer cells from phagocyte attacks due to upregulation of the “don’t eat me” signal CD24. Disruption of DYRK1B reduces the inflammatory secretome, while increasing production of chemoattractants which recruit tumoricidal macrophages. These macrophages can eliminate cancer cells via phagocytosis, enhance the Th1 T cell response, and ultimately restrict tumor growth. (Figure created in BioRender.com).

In a recent study by us [67], we could demonstrate that inhibitors of DYRK1B provide a novel and clinically translatable approach to targeting both the cancer cell compartment and its microenvironment. Genetic ablation or pharmacological inhibition of DYRK1B changed the secretome of PDAC tumor cells to strongly attract macrophages into tumor sites via the overproduction of CCL5, CCL6, CXCL2, and MCSF. In addition, infiltrated macrophages exhibited an M1-like phenotype with reduced inflammatory potential, which subsequently attenuated the development of an inflammatory phenotype of surrounding CAFs while also activating CD4^+^ T cells. Importantly, targeting DYRK1B downregulated the “don’t eat me” signal CD24 on cancer cells, resulting in enhanced tumor cell phagocytosis by macrophages (Fig. 2). The precise molecular mechanism of this interaction remains to be elucidated but, as outlined before, previous work has already firmly linked DYRK1B with the regulation of protein stability [48–50].

Moreover, a small-molecule DYRK1B-directed therapy combined with mTOR inhibition and conventional chemotherapy restricted the growth of established tumors and extended survival in an aggressive murine PDAC mouse model, significantly extending the understanding of DYRK1B as an anti-cancer target. In addition to the aforementioned cell-intrinsic functions of DYRK1B in inducing proliferative quiescence, cell survival, and DNA repair to evade chemotherapy, this kinase also promotes immune evasion, preventing attacks on tumor cells by tumoricidal macrophages. The concept of DYRK1B as a promoter of cancer relapse due to its cell-intrinsic regulation of cell cycle and survival might thus be too narrow. Instead, it additionally appears that DYRK1B exerts potent cell-extrinsic functions in the TME, thereby creating an immunological safe haven and shielding cancer cells from the host immune system.

Pharmacological modulation of DYRK1B thus represents a very promising anti-cancer strategy, acting on multiple layers to control macrophage immunity. In this regard, clinical DYRK1B inhibitors might potentiate immune checkpoint blockade (αPD-1/PD-L1; α-CTLA4) or myeloid-stimulating approaches such as α-CD40 ligands. They could also be co-applied with the adoptive transfer of CAR-modified macrophages to facilitate their infiltration into tumors, prevent M2 polarization, potentiate Th1 immune response, and enhance phagocytosis of CD24-positive tumor cells.

### The prospect of clinical DYRK1B inhibition

So far, a broad range of various DYRK inhibitors has been developed and used in pre-clinical research [113–115]. Prominent examples of DYRK inhibitors are Harmine [116], AZ191 [49], Epigallocatechin 3-gallate (EGCG) [117], or the Leucettinibs [118]. Unfortunately many, if not all, of the identified compounds are not exclusively specific for a given DYRK family member and typically block several DYRK enzymes, particularly at higher concentrations. Two inhibitors are currently in early clinical trials for the treatment of neurological disease in Down Syndrome and/or Alzheimer patients: EGCG (NCT01394796; phase II) and Leucettinib-21 (NCT06206824; phase I [119]). In addition, inhibitors of the closely related CLK family of kinases tend to target DYRKs as well and have proven very promising in preclinical cancer models [120]. Furthermore, a new approach to target DYRK1 enzymes are proteolysis targeting chimeras (PROTACs) [121]. Here, an ATP-competitive DYRK1 inhibitor has been coupled to a Cereblon ligand, resulting in the ubiquitination and the subsequent proteasomal degradation of DYRK1A and DYRK1B [121]. Such approaches will be of high interest for targeting kinase-independent effects of DYRKs such as in metabolic control (see paragraph above).

## Conclusion

Future cancer therapy will have to employ cancer cell-centered therapies (cytotoxic or targeted) in combination with TME-based approaches. This will particularly hold true for stroma-rich solid cancers such as PDAC, where the prognosis still remains poor while the incidence is rising. Here we proposed DYRK1B as an attractive therapeutic target as it promotes cancer growth by simultaneously modulating cell-intrinsic as well as TME aspects, functionally creating a protective niche for malignant cells. Small-molecule DYRK1 inhibitors are now in clinical testing and might therefore be soon applicable to remove this “safe haven” for cancer cells.
